# Dysregulation of Cells Cycle and Apoptosis in Human Induced Pluripotent Stem Cells Chondrocytes Through p53 Pathway by HT-2 Toxin: An *in vitro* Study

**DOI:** 10.3389/fgene.2021.677723

**Published:** 2021-08-04

**Authors:** Yanan Zhang, Huan Liu, Xialu Lin, Feng’e Zhang, Peilin Meng, Sijia Tan, Mikko J. Lammi, Xiong Guo

**Affiliations:** ^1^School of Public Health, Health Science Center of Xi’an Jiaotong University, Xi’an, China; ^2^Key Laboratory of Trace Elements and Endemic Diseases of National Health Commission and Collaborative Innovation Center of Endemic Diseases and Health Promotion in Silk Road Region, Xi’an, China; ^3^Department of Integrative Medical Biology, University of Umeå, Umeå, Sweden

**Keywords:** Kashin–Beck disease, HT-2 toxin, cell cycle, cell apoptosis, p53

## Abstract

Kashin–Beck disease (KBD) mainly damages growth plate of adolescents and is susceptible to both gene and gene–environmental risk factors. HT-2 toxin, which is a primary metabolite of T-2 toxin, was regarded as one of the environmental risk factors of KBD. We used successfully generated KBD human induced pluripotent stem cells (hiPSCs) and control hiPSCs, which carry different genetic information. They have potential significance in exploring the effects of HT-2 toxin on hiPSC chondrocytes and interactive genes with HT-2 toxin for the purpose of providing a cellular disease model for KBD. In this study, we gave HT-2 toxin treatment to differentiating hiPSC chondrocytes in order to investigate the different responses of KBD hiPSC chondrocytes and control hiPSC chondrocytes to HT-2 toxin. The morphology of HT-2 toxin-treated hiPSC chondrocytes investigated by transmission electron microscope clearly showed that the ultrastructure of organelles was damaged and type II collagen expression in hiPSC chondrocytes was downregulated by HT-2 treatment. Moreover, dysregulation of cell cycle was observed; and p53, p21, and CKD6 gene expressions were dysregulated in hiPSC chondrocytes after T-2 toxin treatment. Flow cytometry also demonstrated that there were significantly increased amounts of late apoptotic cells in KBD hiPSC chondrocytes and that the mRNA expression level of Fas was upregulated. In addition, KBD hiPSC chondrocytes presented stronger responses to HT-2 toxin than control hiPSC chondrocytes. These findings confirmed that HT-2 is an environmental risk factor of KBD and that p53 pathway interacted with HT-2 toxin, causing damaged ultrastructure of organelles, accelerating cell cycle in G1 phase, and increasing late apoptosis in KBD hiPSC chondrocytes.

## Introduction

Kashin–Beck disease (KBD) is an endemic articular disease and mainly affects people living in southeast Siberia of Russia, the northern region of North Korea, and the long narrow zone from northeastern to southwestern of China (Yu et al., 2019b; Wang et al., 2020). KBD mainly damages the epiphyseal growth plate and causes necrosis and apoptosis of chondrocytes in the deep zone of the cartilage (Guo et al., 2014). The damaged epiphyseal growth plate and chondrocytes mostly result in short stature and a secondary chronic osteoarthropathy presenting enlarged and deformed joints (Xiong, 2001). It is generally believed that KBD is a gene–environmental risk factor interactive disease (Yu et al., 2016). Although previous studies have tried to establish a KBD disease model using rats (Guan et al., 2013), mice (Yang et al., 1993), chickens (Walser et al., 1988), mini-pigs (Yongmin et al., 1998), and rhesus monkeys (Zhang and Liu, 1989), they have failed to find exactly the same pathological changes with KBD in human. Although articular cartilage damage may be achieved by the models, they do not produce multiple foci in the deep zone but extend to the full depth of cartilage (Wang et al., 2011). In rodents, the growth plates are present in even the aged animals (Roach et al., 2003), indicating a different regulation of chondrogenesis or its maintenance. Moreover, in the areas of human KBD cases, no similar animal cases are found. Hence, the etiological research of KBD has been hampered due to lack of animal and cellular disease models.

Induced pluripotent stem cells (iPSCs) were first generated by transducing mouse fibroblasts with four factors, i.e., c-Myc, Klf4, Oct-3/4, and Sox-2, in 2006 (Takahashi and Yamanaka, 2006). Later, human iPSCs (hiPSCs) were successfully reprogrammed from adult human fibroblasts with the similar methods in 2007 (Takahashi et al., 2007). The hiPSCs have a potential of multiple-lineage differentiation, which is similar to human embryonic stem cells (hESCs). The advantage of the hiPSCs is that they are not involved with ethical issues related to sacrificing embryos for the generation of ESCs (Tsumaki et al., 2015; Liu et al., 2017). Their self-renewal and multiple-lineage differentiation potential provide investigators a wide range of applications, including tissue regeneration, drug screening, and disease modeling. In principle, the hiPSCs can be generated from any human somatic cell type, either healthy individuals or specific patients. Therefore, the hiPSCs can provide perfect a human disease model to study both disease pathology, especially in diseases with specific genetic background, and their response to drugs and toxins (Bellin et al., 2012; Avior et al., 2016). In particular, the hiPSC technology is an optimized choice for generating cell lines of the diseases that are genetically susceptible and for modeling diseases for which affected tissues cannot be easily accessed (Park et al., 2008; Tiscornia et al., 2011). Researchers have established different disease models using iPSCs, including neural developmental disease, Parkinson’s disease, amyotrophic lateral sclerosis, and rheumatoid arthritis (Badger et al., 2014; Myszczynska and Ferraiuolo, 2016; Ardhanareeswaran et al., 2017; Kim et al., 2018; Shahi et al., 2019). Since KBD is a disease that is apparently susceptible to both genetic and environmental risk factors and the affected tissues are difficult to obtain, the hiPSCs generated from KBD could be a perfect choice for a cellular disease model.

It has been revealed that T-2 toxin contamination in cereals is one of the environmental risks of KBD by inhibiting proliferation of chondrocytes, increasing chondrocytes apoptosis, and activating the catabolism of chondrocytes, resulting in proteoglycan degradation in cartilage (Chen et al., 2006; Tian et al., 2012; Guo et al., 2014). Recent studies found that HT-2 toxin was a primary metabolite of T-2 toxin in human chondrocytes with less toxicity than T-2 toxin (Yu et al., 2017a). In addition, HT-2 toxin was detected in rats’ skeletal system after 24 h of T-2 toxin treatment (Yu et al., 2017b). Moreover, *in vitro* study showed that both T-2 toxin and HT-2 toxin caused human chondrocyte apoptosis and autophagy (Yu et al., 2019a). Based on previous studies, it still needs to be explored whether the HT-2 toxin is a direct risk factor of KBD.

p53 pathway is crucial for regulation of cell cycle, DNA damage, and cell apoptosis. Previous studies have reported that p53 expression level was upregulated in KBD chondrocytes compared with healthy chondrocytes (Xiong et al., 2010; Yu et al., 2016; Yang et al., 2017, 2019). Furthermore, p53 and related proteins, including Fas and caspase-3, were activated in human chondrocytes and induced apoptosis after T-2 toxin treatment (Chen et al., 2008). Meanwhile, mice with overexpressed CDK6 and CCND1 presented dwarfism, and dwarfism was relieved in mice with p53 knock-out (Ito et al., 2014). Considering that KBD chondrocytes also have increased apoptosis and dysregulation of cell cycle, and patients with severe KBD (Grade III) suffer from dwarfism, we speculated that p53 pathway might be involved in KBD progression dysregulation of cell cycle and apoptosis of chondrocytes.

To investigate whether HT-2 toxin was a direct environmental risk factor of KBD and its mechanism in dysregulation of cell cycle and apoptosis in KBD chondrocytes, we adopted KBD hiPSCs and control hiPSCs, which carry different genetic information, and we differentiated hiPSCs into hiPSC chondrocytes and then treated the hiPSC chondrocytes and the control hiPSC chondrocytes with HT-2 toxin in order to simulate the progression of KBD. Genes involved in p53 pathway were also analyzed to explore the molecular mechanism of KBD.

## Materials and Methods

### The Human Induced Pluripotent Stem Cell Culture, Chondrocyte Differentiation, and HT-2 Toxin Treatment

The hiPSCs were reprogrammed and cultured as described in submitted paper “The first human induced pluripotent stem cell line of Kashin-Beck disease reveals involvement of heparan sulphate proteoglycan biosynthesis and PPAR pathway in the disease” (*FEBS Journal*, provisionally accepted for publication). Briefly, skin fibroblasts from a healthy person and a KBD patient were reprogrammed to the hiPSCs by transfecting them with Oct-4, Sox-9, and c-Myc through a retrovirus. The disease was diagnosed according to National Diagnostic Criteria for KBD (WS/T 207-2010) in China and confirmed by three orthopedists. Considering the instability of 0~12 populations of the hiPSCs, all hiPSCs used in this study were between 12 and 45 passages. Undifferentiated hiPSCs were seeded on plates coated with BD Matrigel™ and maintained in PSCeasy stem cell culture medium (Cellapy, Beijing, China). Chondrocyte differentiation was carried out following a modified direct protocol (Suchorska et al., 2017). The hiPSCs were seeded in a six-well plate (Corning, New York, NY, USA) and expanded in hiPSC culture medium to about 60~70% confluency. Then the medium was replaced with chondrogenic medium containing low-glucose DMEM/F12 (HyClone, Logan, UT, USA), 10% fetal bovine serum (FBS; HyClone, USA), 1% penicillin/streptomycin (Sigma-Aldrich Corp., St. Louis, MO, USA), 10 ng/ml of FGF-2 (PeproTech, Cranbury, NJ, USA), 10 ng/ml of BMP-4 (PeproTech, USA), and 10 ng/ml of PDGF-BB (PeproTech, USA). After the 7-day differentiation period, the medium was replaced with chondrogenic medium supplemented with low-glucose DMEM/F12 (HyClone, USA), 10% FBS (HyClone, USA), 1% penicillin/streptomycin (Sigma, USA), 1% insulin-transferrin-selenium (ITS), 50 μM of l-proline, 50 μM of ascorbic acid, 1 mM of sodium pyruvate, 10^−7^ M of dexamethasone, and 10 ng/ml of TGF-beta 3 (PeproTech, USA). During the whole differentiation period, the medium was changed every 2–3 days. The hiPSC differentiated chondrocytes were named hiPSC chondrocytes. For the cells in HT-2 treatment group (0–500 ng/ml), the hiPSCs were cultured using the normal differentiation protocol. After the 7-day differentiation, HT-2 toxin was given to the hiPSC chondrocytes until the end of the differentiation.

### Immunofluorescence Staining

Immunofluorescence staining was adopted to assess the expression level of chondrocyte markers type I collagen (Col1), type II collagen (Col2), and type X collagen (Col10). After differentiation of the cells at days 7, 14, and 21, the cells were fixed in 4% paraformaldehyde, followed by permeabilization with 0.5% Triton X-100 in phosphate-buffered saline (PBS). After being washed, the cells were blocked with 1% bovine serum albumin (BSA) in 0.3 M of glycine and incubated with primary antibody against Col1 (AB34710 at 1:500 dilution, Abcam, Cambridge, United Kingdom), Col2 (AB34712 at 1:250 dilution, Abcam, United Kingdom), and Col10 (AB49945 at 1:1,000 dilution, Abcam, United Kingdom) overnight at 4°C. An Alexa Fluor® 488-conjugated goat anti-rabbit IgG and an Alexa Fluor® 594-conjugated goat anti-mouse IgG were used as the secondary antibody. The cellular nuclei were counter-stained with DAPI (Sigma, USA). The immunofluorescence was observed with a Nikon-Ti inverted fluorescence microscope (Nikon, Tokyo, Japan).

### MTT Cytotoxicity Assay

The cell viability after HT-2 toxin treatment was assessed by MTT assay, which is based on the metabolic activity of mitochondria. The metabolic activity and content of the cells were assumed to remain the same during the treatment. In order to assess the cytotoxicity of HT-2 toxin on the hiPSC chondrocytes, they were differentiated for 7 days, then seeded in a 96-well plate, and allowed to attach overnight. Then HT-2 toxin (0–20 ng/ml) was added until the end of differentiation. Four hours before the end of HT-2 toxin treatment, 20 μl of the 5 mg/ml MTT (Amresco, Solon, OH, USA) stock solution was added and incubated for 4 h. Then the medium was carefully removed, and 150 μl of dimethyl sulfoxide (Sigma, USA) was added in each well to solve the insoluble formazan crystals. The absorbance of each well was measured in a multiplate reader (Infinite M200, Tecan, Männedorf, Switzerland) at a wavelength of 490 nm. Each experiment was repeated at least three times.

### RNA Extraction and qRT-PCR Analysis

Total RNA was extracted with TRIzol (Invitrogen, Carlsbad, CA, USA) following the standard protocol. The concentration of RNA was determined using a spectrophotometer (Nano Drop Technologies, Wilmington, DE, USA). Reverse transcription was performed with 500 ng of total RNA using PrimeScript™ RT Master Mix (Takara Bio, Mountain View, CA, USA). qRT-PCR for GADPH, COL2A1, p53, p21, CDK6, CCND1, Fas, CASP3, and CASP9 was conducted using SYBR green system on StepOne Plus real time cycler (Bio-Rad, Hercules, CA, USA) for 40 cycles of amplifications, consisting of duration at 95°C for 10 s and extension at 60°C for 30 s. The relative mRNA expression level was calculated using 2^−∆∆Ct^ method and normalized to GADPH. The control hiPSC chondrocytes without HT-2 toxin treatment were used as a reference for COL2A1, p53, p21, CDK6, CCND1, Fas, CASP3, and CASP9. Gene-specific primers used in this study for qRT-PCR are shown in Table 1.

**Table 1:** Gene-specific primers of qRT-PCR reaction.

Gene	Primer sequence (5'-3')
GAPDH	F: GCACCGTCAAGGCTGAGAAC
	P: TGGTGAAGACGCCAGTGGA
COL2A1	F: AGACTGGCGAGACTTGCGTCTA
	R: ATCTGGACGTTGGCAGTGTTG
P53	F: TCGAGATGTTCCGAGAGCTGAAT
	R: GTCTGAGTCAGGCCCTTCTGTCTT
P21	F: CATGTGGACCTGTCACTGTCTTGTA
	R: GAAGATCAGCCGGCGTTTG
CDK6	F: GTGACCAGCAGCGGACAAATAA
	R: AGCAAGACTTCGGGTGCTCTGTA
CCND1	F: GTGCATCTACACCGACAACTCC
	R: GTTCCACTTGAGCTTGTTCACC
FAS	F: CAAGGGATTGGAATTGAGGAAG
	R: CTGGAGGACAGGGCTTATGG
CASP3	F: GACTCTGGAATATCCCTGGACAACA
	R: AGGTTTGCTGCATCGACATCTG
CASP9	F: GCACAGGGTCTGCTCTTTCTCT
	R: CCACGGCATTCATCTGTCC

### Transmission Electron Microscopy

After HT-2 toxin treatment, the cells were digested and fixed with 2.5% glutaraldehyde for more than 24 h followed by fixation with 1% osmium tetroxide for another 2 h. Specimens were dehydrated with ethanol and butanol mixtures following standard protocol (Tizro et al., 2019) and observed under HITACHIH-7650 transmission electron microscope (Hitachi, Tokyo, Japan).

### Flow Cytometry Assay

**Figure 1:**
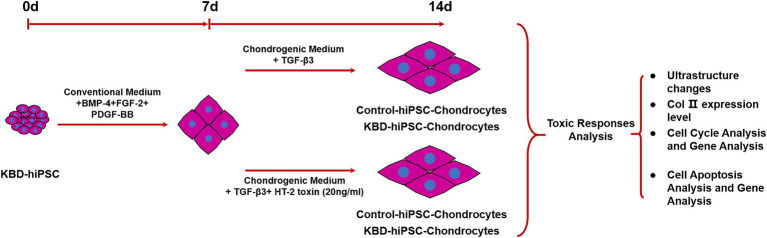
Study scheme of this study. Both the control hiPSCs and the KBD hiPSCs were differentiated into chondrocytes. In order to test the adverse effect of HT-2 toxin on the chondrogenesis of the control hiPSCs and the KBD hiPSCs, the hiPSC chondrocytes differentiated for 7 days were exposed to HT-2 toxin (20 ng/ml) for 7 days before ultrastructure changes, chondrogenesis, cell cycle, and cell apoptosis were analyzed. hiPSCs, human induced pluripotent stem cells; KBD, Kashin–Beck disease.

Cells were harvested after treatment following the study protocol (Figure 1). For cell cycle analysis, the cells were fixed with precooling ethanol and stained with propidium iodide (PI), and fluorescence was detected using FACSCalibur (BD Biosciences, San Jose, CA, USA) at wavelength of 488 nm. For cell apoptosis, APC Annexin V Apoptosis detection kit with PI (BioLegend, San Diego, CA, USA) was used to fix and stain with Annexin V–fluorescein isothiocyanate (FITC) and PI according to standard protocol. Then samples were detected at FACSCalibur. BD CellQuest software was used for cell cycle and cell apoptosis analyses.

### Statistical Analysis

Statistical analysis was performed with GraphPad Prism software (version 7). The statistical significance among HT-2 toxin treatment groups was assessed by one-way ANOVA with *post hoc* Scheffe’s test. All results in this study were obtained from at least three independent repeats. Significance was set at *p* < 0.05.

## Results

### The Human Induced Pluripotent Stem Cells Could Differentiate Into Chondrocytes

**Figure 2:**
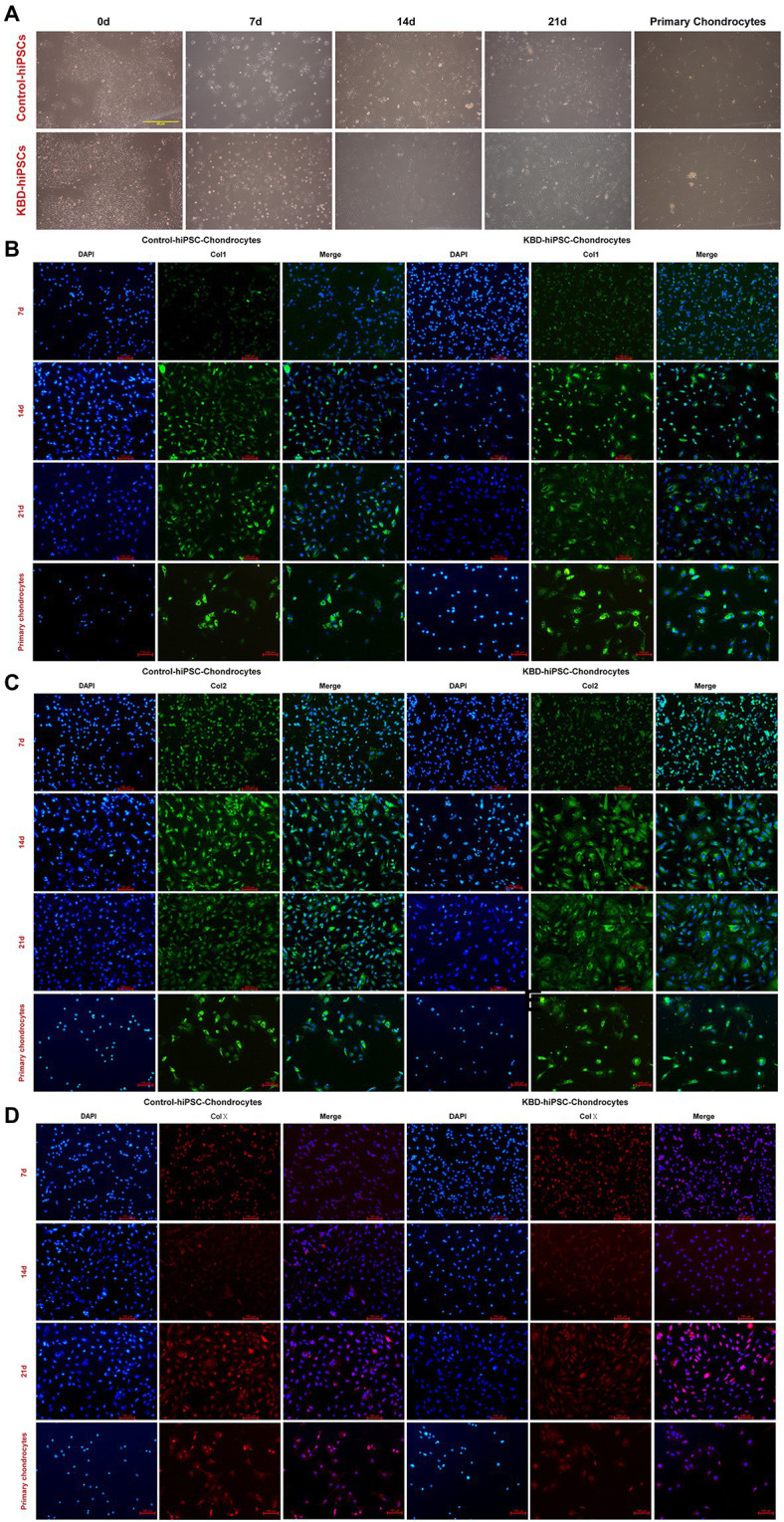
Both the control hiPSCs and the KBD hiPSCs could differentiate to chondrocytes. **(A)** Cell morphological changes under optical microscope of the hiPSCs during the process of chondrogenesis. **(B–D)** Immunofluorescence staining of the hiPSCs at days 7, 14, and 21 during chondrogenic differentiation. Both the differentiated control hiPSCs and the KBD hiPSCs could express Col1 **(B)**, Col2 **(C)**, and Col10 **(D)**. hiPSCs, human induced pluripotent stem cells; KBD, Kashin–Beck disease.

In this study, the hiPSCs were differentiated to the hiPSC chondrocytes following standard protocol of previous studies (Suchorska et al., 2017). After 14 days of differentiation, the cell morphology changed (Figure 2A). Immunofluorescence staining results showed that both the control hiPSC chondrocytes and the KBD hiPSC chondrocytes expressed Col1 (Figure 2B), Col2 (Figure 2C), and Col10 (Figure 2D) after 7-day differentiation, and even stronger fluorescence was present after 14 days of differentiation. Considering that the hiPSCs expressed Col2 after 7-day differentiation, we assume that the 7-day differentiated cells were chondrocytes of early stage. Therefore, we shortened the differentiation period to 14 days in the following experiments.

### Cytotoxic Effect of HT-2 Toxin on the Human Induced Pluripotent Stem Cell Chondrocytes

**Figure 3:**
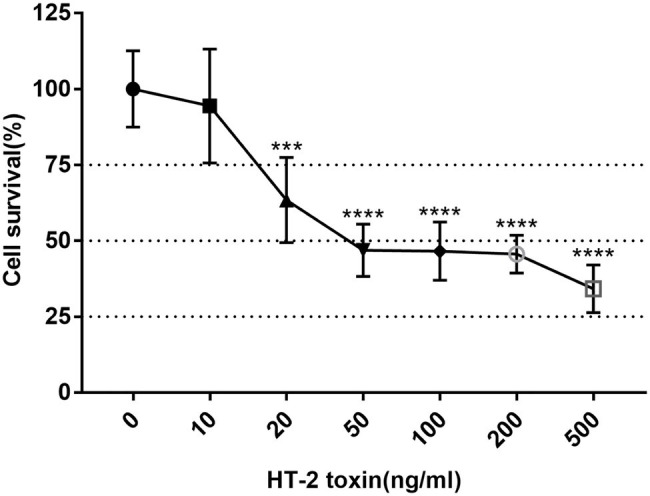
Cell viability of the KBD hiPSC chondrocytes after 7-day exposure of HT-2 toxin. Reported values are the mean ± SD of three independent experiments. ^***^p < 0.001, a significant difference between the treatment and control group (0 ng/ml); ^****^p < 0.0001; *n* = 3. KBD, Kashin–Beck disease; hiPSCs, human induced pluripotent stem cells.

To determine the suitable concentration of HT-2 toxin for the hiPSC chondrocytes, MTT assay was applied to observe the toxicity of HT-2 toxin on the hiPSCs. The hiPSCs were differentiated for 7 days and exposed to HT-2 toxin at different concentrations ranging from 0 to 500 ng/ml for 7 days. There was a clear dose-dependent effect among HT-2 toxin concentration and cell viability (Figure 3). No significant difference was observed between the control group (0 ng/ml) and 10 ng/ml HT-2 toxin treatment group (Figure 3). The administration of 20 ng/ml of HT-2 toxin treatment resulted in lower cell viability as compared with the control group (0 ng/ml), and the difference was statistically significant (*p* < 0.01; Figure 3). When the concentration of HT-2 toxin was up to 50 ng/ml, the cell viability was dramatically decreased (46.86%) and the difference was statistically significant (*p* < 0.01; Figure 3). Conclusively, 20 ng/ml HT-2 toxin was considered to be effective, while not too toxic, for the hiPSC chondrocytes with 63.40% cell viability. Therefore, in the following analyses, the hiPSC chondrocytes were treated with 20 ng/ml HT-2 toxin.

### HT-2 Toxin Inhibited Human Induced Pluripotent Stem Cell Differentiation Into Chondrocytes

**Figure 4:**
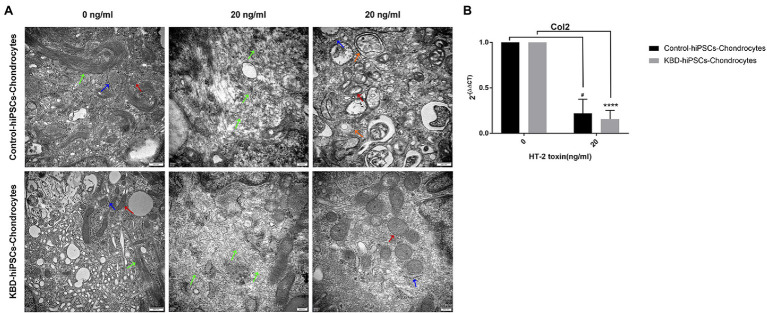
HT-2 toxin inhibited the hiPSCs differentiating to chondrocytes and downregulated Col2 expression. **(A)** TEM showed that ultrastructure changes in the hiPSC chondrocytes after HT-2 toxin exposure; red arrowheads indicate mitochondria, green arrowheads indicate microtubules, blue arrowheads indicate endoplasmic reticulum, and orange arrowheads indicate autophagic vacuole. **(B)** Col2 mRNA expression was downregulated after treatment with HT-2 toxin in the hiPSC chondrocytes. ^#^A difference between HT-2 toxin treatment (20 ng/ml) and non-toxin treatment of the control hiPSC chondrocytes, *p* < 0.05. ^****^A significant difference between the HT-2 toxin-treated and non-treated KBD–hiPSC chondrocytes, *p* < 0.0001. hiPSCs, human induced pluripotent stem cells; TEM, transmission electron microscopy; KBD, Kashin–Beck disease.

We could observe ultrastructural changes in the hiPSC chondrocytes by transmission electron microscopy (TEM). Microtubules are normal intracellular structures of the cells, and bundles of microtubules were present in the control cells without HT-2 toxin treatment. However, after 7-day treatment with HT-2 toxin, the arrangements of microtubules in the control hiPSC chondrocytes and the KBD hiPSC chondrocytes were both disordered. Meanwhile, swollen mitochondria with vacuolar degeneration were found at increased abundance in the HT-2-treated KBD hiPSC chondrocytes and the control hiPSC chondrocytes. In addition, both the control hiPSC chondrocytes and the KBD hiPSC chondrocytes had presence of dilated endoplasmic reticulum. However, no apoptotic-like bodies were found in the HT-2-treated KBD hiPSC chondrocytes and the control hiPSC chondrocytes (Figure 4A). After HT-2 toxin treatment, mRNA expression level of Col2 was downregulated in the hiPSC chondrocytes (Figure 4B). These results indicated that HT-2 toxin could apparently inhibit both the control hiPSC and KBD hiPSC differentiation into the chondrocytes. However, there was no significant difference between the HT-2 toxin-treated control hiPSC chondrocytes and the KBD hiPSC chondrocytes (*t* = 0.585, *p* = 0.600).

### Cell Cycle Was Accelerated After HT-2 Toxin Treatment of the Human Induced Pluripotent Stem Cell Chondrocytes

**Figure 5:**
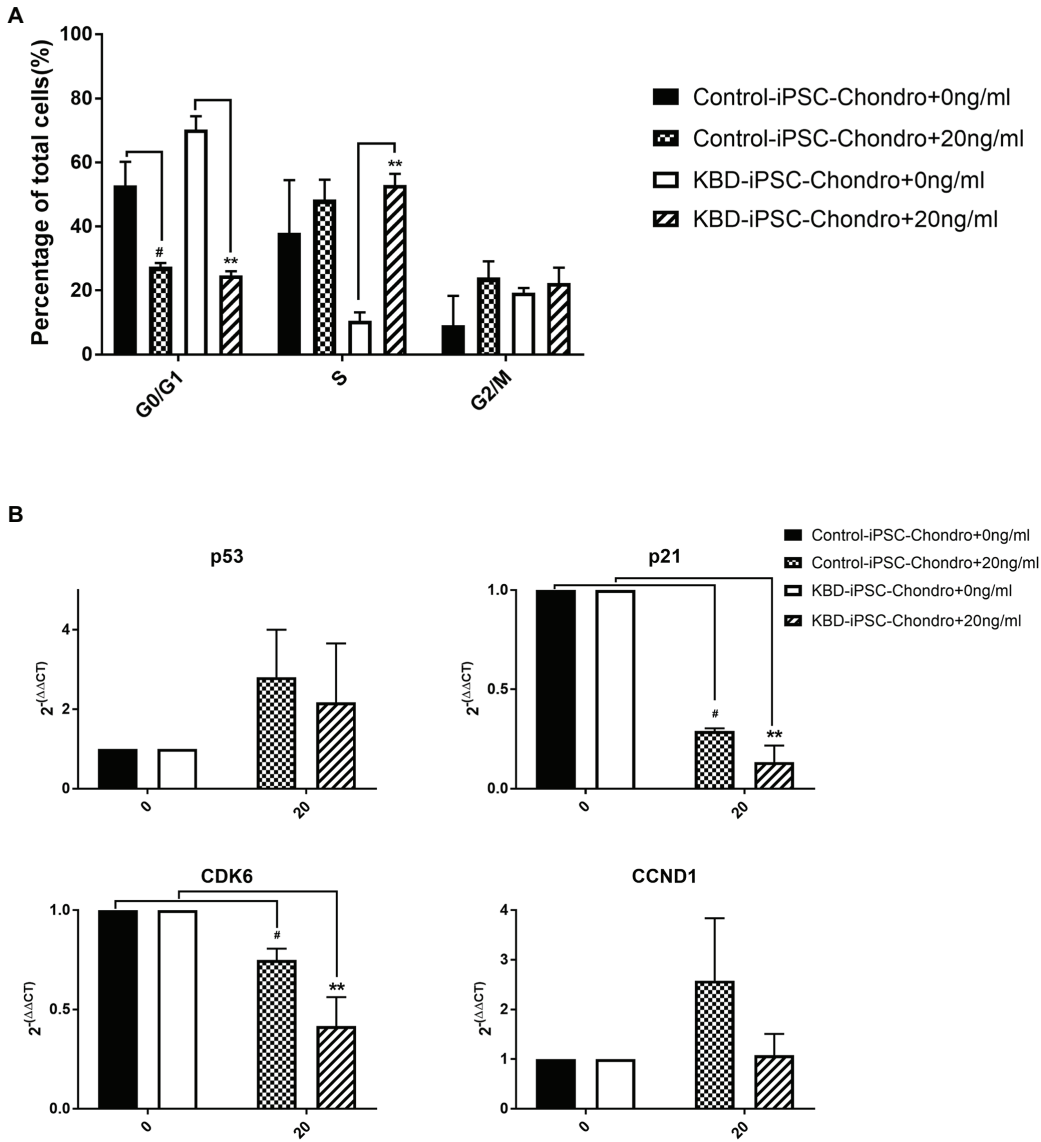
**(A)** Cell cycle was accelerated at the G1 to S phase after HT-2 toxin exposure. **(B)** p53 appeared upregulated in both the KBD hiPSC chondrocytes and the control hiPSC chondrocytes after HT-2 toxin exposure. However, the differences were not statistically significant. p21 and CDK6 were both downregulated in the KBD hiPSC chondrocytes and the control hiPSC chondrocytes after HT-2 toxin treatment, and the KBD hiPSC chondrocytes presented a lower expression level. ^#^A significant difference between the HT-2 toxin-treated and non-treated control hiPSC chondrocytes, *p* < 0.05. ^**^A significant difference between the HT-2 toxin-treated and non-treated KBD hiPSC chondrocytes, *p* < 0.01. hiPSCs, human induced pluripotent stem cells; KBD, Kashin–Beck disease.

In order to confirm the effects of HT-2 toxin on the cell cycle in the hiPSC chondrocytes, flow cytometry analysis was performed. The results showed that HT-2 toxin significantly induced the cell cycle acceleration in both the control hiPSC chondrocytes and the KBD hiPSC chondrocytes (Figure 5A). After treatment with HT-2 toxin, the cell population in G0/G1 phase was significantly decreased in the control hiPSC chondrocytes (53.40 ± 6.55% vs. 27.46 ± 1.11%, *p* < 0.05) and the KBD hiPSC chondrocytes (70.25 ± 4.17% vs. 24.75 ± 1.29%, *p* < 0.01). While cell population in S phase was significantly increased in the KBD hiPSC chondrocytes (10.49 ± 2.69% vs. 52.93 ± 3.52%, *p* < 0.01), there was no significant difference in the number of the cells in G2/M phase in either the control hiPSC chondrocytes or the KBD hiPSC chondrocytes treated with HT-2 toxin (Figure 5A). Overall, we found that HT-2 toxin decreased the relative number of the cell in G0/G1 phase cells and that HT-2 toxin produced more significant effects on KBD hiPSC chondrocytes. The qRT-PCR analysis suggested an apparent upregulation of p53 by HT-2 toxin, while the differences between the HT-2 toxin treatment and the control were not statistically significant. The mRNA expression levels of p21 and CDK6 were both downregulated in the KBD hiPSC chondrocytes and the control hiPSC chondrocytes after HT-2 toxin treatment (Figure 5B). Overall, our results presented that HT-2 toxin led to more differentiation of hiPSC chondrocytes to go into mitosis instead of chondrogenesis.

### Cell Apoptosis Was Increased After HT-2 Toxin Treatment of Human Induced Pluripotent Stem Cell Chondrocytes

**Figure 6:**
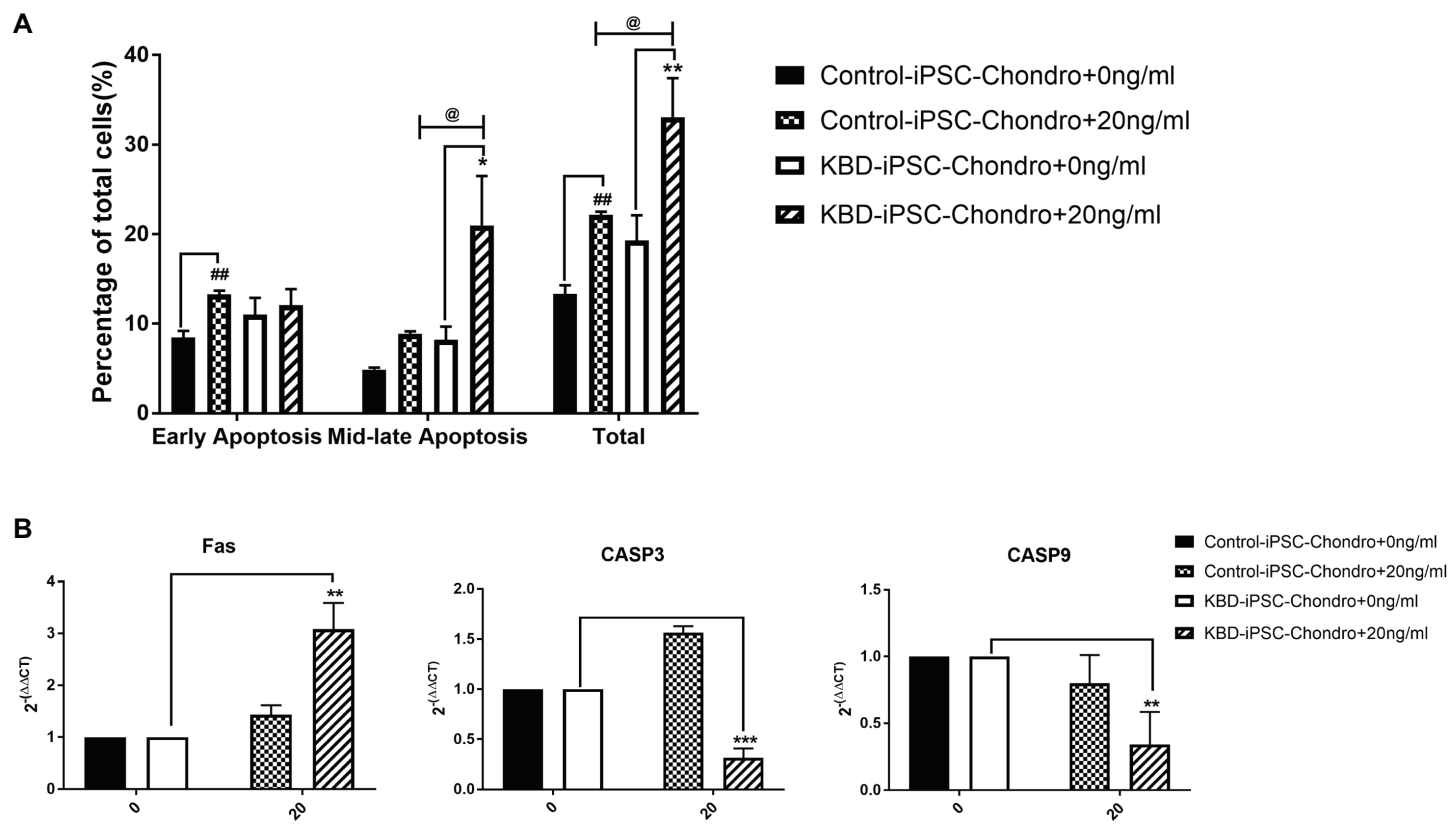
**(A)** HT-2 toxin caused mid-late apoptosis in the hiPSC chondrocytes. **(B)** Fas was significantly upregulated in the KBD hiPSC chondrocytes after HT-2 toxin exposure; CASP3 and CASP9 were significantly downregulated in the KBD hiPSC chondrocytes. ^##^A significant difference between the HT-2 toxin-treated and non-treated control hiPSC chondrocytes, *p* < 0.01. ^*^A significant difference between the HT-2 toxin-treated and non-treated KBD–hiPSC chondrocytes, ^**^p < 0.01 and ^***^p < 0.001. ^@^A significant difference between HT-2 toxin-treated control hiPSC chondrocytes and KBD hiPSC chondrocytes. hiPSCs, human induced pluripotent stem cell; KBD, Kashin–Beck disease.

Flow cytometry analysis of cell apoptosis showed that significantly increased level of early apoptosis was found only in the control hiPSC chondrocytes after HT-2 toxin treatment (HT-2 treated 13.28 ± 0.41% vs. non-treated 8.49 ± 0.71%, *p* < 0.01; Figure 6A), while the percentage of mid-late apoptotic cells was significantly increased in HT-2 toxin-treated group of KBD hiPSC chondrocytes (HT-2 treated 21.00 ± 5.49% vs. non-treated 8.22 ± 1.46%, *p* < 0.05). Meanwhile, the total apoptotic cells in HT-2 toxin treatment of KBD hiPSC chondrocytes was significantly higher than those in control hiPSC chondrocytes (HT-2-treated KBD hiPSC chondrocytes 33.08 ± 4.35% vs. HT-2-treated control hiPSC chondrocytes 22.18 ± 0.34%, *p* < 0.05). Real time RT-PCR results showed that mRNA expression level of Fas was significantly increased after HT-2 toxin exposure in the KBD hiPSC chondrocytes (*p* < 0.01), while CASP3 and CASP9 mRNA expression levels were downregulated (Figure 6B).

## Discussion

T-2 toxin is a type A mycotoxin, which is considered to be an environmental risk factor of KBD through causing damage in developing cartilage. It is also well-known that HT-2 toxin is a primary metabolite of T-2 toxin (Wu et al., 2020). There is a need for a disease model for KBD, which could reveal the pathologic processes of early chondrogenic differentiation. In this study, we used healthy and KBD hiPSC cell lines to try to find out the differences in response to environmental risk factor of KBD, HT-2 toxin. Indeed, we could observe differential expression of genes, including p21, CDK6, Fas, CASP3, and CASP9, which responded differentially to HT-2 toxin treatment.

In order to compare the toxicity of HT-2 toxin on differentiating control hiPSC chondrocytes and KBD hiPSC chondrocytes, we first adopted TEM to observe the ultrastructural changes of cellular organelles after HT-2 toxin exposure. Disordered arrangement of microtubules, swelling, and vacuolar degeneration of mitochondria and dilated rough endoplasmic reticulum were observed in both the control hiPSC chondrocytes and the KBD hiPSC chondrocytes after HT-2 toxin exposure. This finding is consistent with previous studies on human chondrocytes cell line C28/I2 (Lei et al., 2017). Necrosis and apoptosis are typical features in deep zones of KBD cartilage. Although at the moment we cannot define why there was more apoptosis in the KBD hiPSC chondrocytes after HT-2 treatment, it is not surprising based on the hypothesis that T-2 and HT-2 toxins would be predisposing factors for KBD changes in chondrocytes. Wide-scale gene expression assays investigating the effects of HT-2 toxin on the KBD hiPSC chondrocytes would be the next endeavor to investigate the regulatory mechanisms leading to KBD.

We found that Col2 mRNA expression level was significantly downregulated after HT-2 toxin treatment in both the control hiPSC chondrocytes and the KBD hiPSC chondrocytes in comparison with their non-treated cell cultures. This indicated that HT-2 toxin could inhibit the chondrogenesis in both the control hiPSC chondrocytes and the KBD hiPSC chondrocytes.

**Figure 7:**
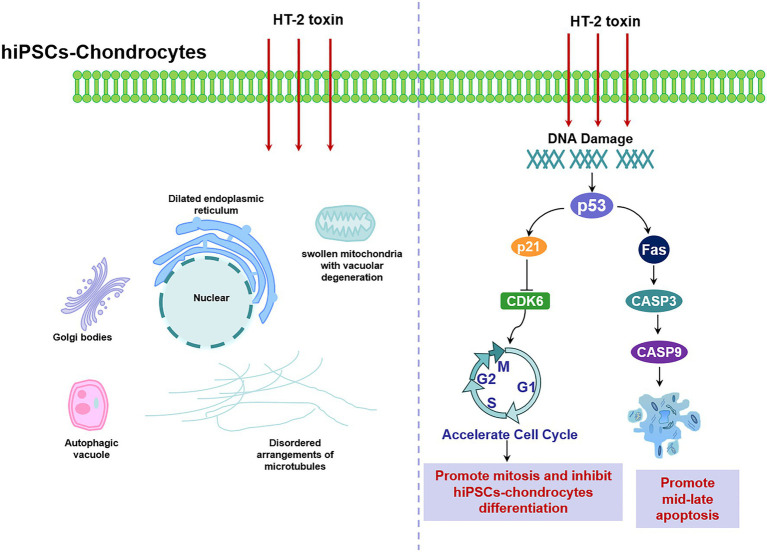
Schematic illustration of HT-2 toxin damaged the differentiating hiPSC chondrocytes by damaging cellular ultrastructure, dysregulating cell cycle, and increasing cell apoptosis through p53. hiPSC, human induced pluripotent stem cell.

Cell cycle regulation plays a critical role in chondrocytes’ proliferation and differentiation. Previous studies have reported that there was dysregulation of cell cycle in KBD chondrocytes, that T-2 toxin could induce cell cycle arrest in human chondrocytes and human liver cells, and that p53 and p21 mRNA expression levels were upregulated (Lei, 2017; Fatima et al., 2018). However, our results revealed that HT-2 toxin could accelerate cell cycle in both the differentiating KBD hiPSC chondrocytes and the control hiPSC chondrocytes. Cell cycle regulation-related genes p21 were significantly downregulated, and cell cycle kinase CDK6 was downregulated. There were no significant differences on p53 mRNA expression level after HT-2 toxin exposure. The main reason behind this may be that the cell sources of previous studies were somatic cells, while our study used the differentiating hiPSC chondrocytes. In the hiPSCs or the ESCs, activation of p53 will lead to suppressed Nanog expression and start differentiation of the hiPSCs (Fu et al., 2020). We started HT-2 toxin treatment after 7 days of differentiation when some cells were still in the early stage of differentiation and had properties of stem cells. This may produce differing effects on the mRNA expression level of p53, p21, and CDK6 (Figure 7).

Chondrocyte apoptosis is increased in KBD compared with healthy ones (Wu et al., 2019). Therefore, we also investigated whether HT-2 toxin could induce apoptosis in the hiPSC chondrocytes. After treatment with HT-2 toxin, the percentage of the mid-late apoptotic cells was significantly increased in the KBD hiPSC chondrocytes, and there was a significant difference between the HT-2 toxin-treated control hiPSC chondrocytes and the KBD hiPSC chondrocytes (Figure 7). mRNA expression levels of the apoptotic regulation genes including Fas, CASP3, and CASP9 were tested. We found that Fas was upregulated only in the KBD hiPSC chondrocytes, while CASP3 and CASP9 were downregulated. Previous studies on somatic cells reported that HT-2 toxin could lead to apoptosis of mammalian reproductive cells with upregulation of p53, CASP3, and CASP9 (Zhang et al., 2019). The gene expression levels were consistent to our findings, and several reasons could explain the inconsistency. First, in our study, HT-2 toxin exposure in the hiPSC chondrocytes was given for 7 days with low concentration, and the process can be considered as chronic toxicity. However, apoptosis is like an early stage of cell death. After 7-day HT-2 toxin treatment, most cells were in the late stage of cell death, which made it hard to find the gene expression level of apoptosis. Second, we found no chromatin changes and apoptotic-like bodies under TEM in the HT-2 toxin-treated hiPSC chondrocytes. We speculate that HT-2 toxin may cause necrocytosis in the differentiating hiPSC chondrocytes. Last, our results indicate that T-2/HT-2 toxin may not be the only environmental risk factors of KBD and that multiple environmental risk factors work together and lead to chondrocyte damage.

In this study, we differentiated human iPSCs to chondrocytes and then investigated the effects of HT-2 toxin on the differentiating hiPSC chondrocytes. We found that HT-2 toxin could damage both the differentiating control hiPSC chondrocytes and the KBD hiPSC chondrocytes by damaging the cellular ultrastructure, dysregulating cell cycle, and increasing cell apoptosis. Meanwhile, the genes involved in p53 pathway were significantly changed, indicating that p53 pathway might be a susceptible pathway, which interacts with HT-2 toxin.

## Data Availability Statement

The original contributions presented in the study are included in the article, further inquiries can be directed to the corresponding authors.

## Ethics Statement

The studies involving human participants were reviewed and approved by Human Ethics Committee of Xi’an Jiaotong University. The patients/participants provided their written informed consent to participate in this study.

## Author Contributions

XG and ML designed the study. YZ, HL, and XL carried out experiments and data acquisition. YZ, FZ, PM, and ST conducted data analysis and interpretation. YZ drafted the manuscript. All authors contributed to the article and approved the submitted version.

## Conflict of Interest

The authors declare that the research was conducted in the absence of any commercial or financial relationships that could be construed as a potential conflict of interest.

## Publisher’s Note

All claims expressed in this article are solely those of the authors and do not necessarily represent those of their affiliated organizations, or those of the publisher, the editors and the reviewers. Any product that may be evaluated in this article, or claim that may be made by its manufacturer, is not guaranteed or endorsed by the publisher.
